# Topical calcineurin inhibitors for atopic dermatitis are not associated with the development of subsequent lymphoma: A pharmacovigilance study using the FAERS database (2014-2025)

**DOI:** 10.1016/j.jdin.2026.01.002

**Published:** 2026-01-27

**Authors:** Domenico Arcuri, Amina Moustaqim-Barrette, Renata Sava, Ivan V. Litvinov

**Affiliations:** a St. Mary's Research Centre, Montreal, Quebec; b Department of Family Medicine, McGill University, Montréal, Quebec; c Department of Specialized Medicine (Dermatology), St. Mary’s Hospital Centre, St. Mary’s Research Centre, Montreal, Quebec

**Keywords:** adverse, adverse drug event, adverse drug reaction, adverse event, atopic dermatitis, azathioprine, Bayesian Analysis, calcineurin inhibitor, cancer, cellcept, crisaborole, cyclosporin, data mining, dermatology, desonide, drug, dupilumab, dupixent, EBGM, eczema, elidel, Empiric Bayesian Geometric Mean, epidemiology, Eucrisa, FAERS, general dermatology, gengraf, halobetasol, immunosuppression, JAK inhibitor, Janus kinase inhibitor, lymphoma, medical dermatology, medication, methotrexate, mycophenolate, neoral, oncology, opzelura, pharmacovigilance, pimecrolimus, protopic, reporting odds ratio, rinvoq, ROR, ruxolitinib, tacrolimus, topical, upadacitinib

*To the Editor:* Tacrolimus ointment and pimecrolimus cream are topical calcineurin inhibitors (TCI) used in the management of atopic dermatitis/eczema. Both medications carry a black box warning for lymphoma in the USA, in turn affecting patient adherence.^1^ In contrast, Health Canada removed the black box warning for topical pimecrolimus in 2019 and for topical tacrolimus in 2021. Pharmacovigilance studies, which conduct postmarket surveillance of adverse drug reactions (ADRs), are an excellent strategy in helping investigators identify safety signals related to particular drug-ADR pairings in the real-world setting.^2^ The FDA Adverse Event Reporting System (FAERS) is a US government-sponsored database which provides investigators with access to millions of clinician-reported drug-ADR pairings, thereby facilitating comprehensive identification of potentially clinically-significant safety signals. This case-controlled study analyzes the FAERS database to determine whether topical calcineurin inhibitor usage is associated with significant lymphoma-related safety signals.

Nonvalidated reports made between January 2014 and March 2025 were downloaded from the FAERS Quarterly Data Extract Files website (https://fis.fda.gov/extensions/FPD-QDE-FAERS/FPD-QDE-FAERS.html) for further analysis. Our study retrieved a total of 56,093,627 ADRs from 17,448,160 de-duplicated cases. Unfortunately, FAERS database analysis does not allow for proper accounting of drug–drug related interactions during ADR reporting. Additionally, data is not validated prior to submission and duplicate reports, alongside reporting bias, may be present. We utilized 2 statistical methods, the reporting odds ratio (ROR) and Empirical Bayesian Geometric Mean (EBGM), in order to identify any potential lymphoma-related safety signals. The EBGM is preferred over the simpler ROR as it is more robust in its analysis of rarer ADRs like lymphoma. A total of 11 medications used in the treatment of atopic dermatitis were assessed. Tacrolimus and pimecrolimus search queries were ran twice to better determine whether explicit specification of dosage form or administration route would influence the formation of a safety signal. With similar intent, aggregate data was sub-stratified by “any indication” versus “atopic dermatitis” prior to statistical analysis.

As shown in Table I, ROR analysis revealed statistically significant lymphoma signals for most assessed drugs, TCIs included. Again, this finding is likely due to the tendency of the ROR to overestimate the risk of rarer ADRs. Table II demonstrates significant EBGM values including azathioprine, mycophenolate mofetil, methotrexate, and all formulations of tacrolimus. Methotrexate is used to treat certain lymphomas. Treatment failure or disease progression of lymphoma while on methotrexate may be classified as an ADR by certain clinicians, explaining its elevated EBGM in our analysis. Nevertheless, topical tacrolimus and pimecrolimus did not demonstrate any significant lymphoma-safety signals. Furthermore, the EBGM value for all formulations of tacrolimus was not statistically significant when specifying atopic dermatitis as an indication.

**Table I:** Drug-lymphoma reporting odds ratio (ROR) analysis. The ROR is the ratio of the odds of a certain drug-ADR pair occurring compared to the odds of that same reaction occurring with all other drugs. Confidence intervals are shown in brackets besides the ROR value. Statistically significant values are bolded. Significance is attained if the lower bound of the confidence interval is > 1. ROR values are listed for drug-lymphoma ADR pairings for either “All” or “Atopic Dermatitis” specific indications. Bactroban serves as a negative control. “All Formulations” for either tacrolimus or pimecrolimus indicate a search query which included ADRs associated with both systemic and topical forms of these drugs. Systemic form of medications is often given to solid organ transplant recipients to achieve immunosuppression. “Topical” indicates a search query only assessing ADRs for topical formulations of either pimecrolimus or tacrolimus

Drug	ROR all indications	ROR atopic dermatitis
Azathioprine	25.9 (24.9, 26.9)	0.32 (0.23, 0.44)
Crisaborole (topical)	0.17 (0.04,0.69)	0.09 (0.01, 0.61)
Cyclosporin	17.1 (16.1, 18.1)	0.75 (0.58, 0.97)
Desonide	7.58 (4.56, 12.6)	4.53 (2.36, 8.73)
Halobetasol propionate	3.79 (1.42, 10.12)	1.89 (0.47, 7.58)
Methotrexate	19.0 (18.6, 19.4)	0.08 (0.06, 0.11)
Mycophenolate mofetil	18.2 (17.6, 18.9)	0.16 (0.12, 0.23)
Pimecrolimus (all formulations)	6.01 (4.09, 8.83)	3.23 (1.91, 5.45)
Pimecrolimus (topical)	5.32 (3.39, 8.35)	2.79 (1.5, 5.19)
Ruxolitinib (topical)	1.86 (0.88, 3.9)	0.79 (0.26, 2.47)
Tacrolimus (all formulations)	19.7 (19.0, 20.5)	0.19 (0.13, 0.27)
Tacrolimus (topical)	12.5 (9.92, 15.9)	4.81 (3.29, 7.01)
Upadacitinib	4.17 (3.7, 4.6)	1.31 (1.08, 1.59)
Negative control: Bactroban	0.32 (0.22, 0.47)	0

*ADR*, Adverse drug reaction; *ROR*, reporting odds ratio.

**Table II:** Drug-lymphoma Empirical Bayesian Geometric Mean (EBGM) values. EBGM analysis uses Gamma-Poisson shrinker required to accurately assess statistical significance for rare ADRs. Statistically significant values are bolded. Significance is attained if EBGM values ≥ 2.0. EBGM values are listed for drug-lymphoma ADR pairings for either “All” or “Atopic Dermatitis” specific indications. Bactroban serves as a negative control. “All Formulations” for either tacrolimus or pimecrolimus indicates a search query which included ADRs associated with both systemic and topical forms of these drugs. “Topical” indicates a search query only assessing ADRs for topical formulations of either pimecrolimus or tacrolimus

Drug	EBGM all indications	EBGM atopic dermatitis
Azathioprine	4.4	5.9 × 10^-2^
Crisaborole (topical)	5.5 × 10^-4^	3.7 × 10^-2^
Cyclosporin	1.46	6.7 × 10^-2^
Desonide	5.0 × 10^-4^	3.1 × 10^-2^
Halobetasol propionate	8.43 × 10^-5^	5.06 × 10^-5^
Methotrexate	121	6.8 × 10^-1^
Mycophenolate mofetil	10.2	1.0 × 10^-1^
Pimecrolimus (all formulations)	1.8 × 10^-3^	1.0 × 10^-3^
Pimecrolimus (topical)	1.1 × 10^-3^	6.2 × 10^-4^
Ruxolitinib (topical)	4.8 × 10^-4^	2.4 × 10^-4^
Tacrolimus (all formulations)	7.12	7.6 × 10^-2^
Tacrolimus (topical)	6.3 ×10^-3^	2.5 × 10^-3^
Upadacitinib	4.2 × 10^-1^	1.3 × 10^-1^
Negative control: Bactroban	3.4 × 10-3	1.2 × 10-4

*ADRs*, Adverse drug reactions; *EBGM*, Empirical Bayesian Geometric Mean.

Analysis of real-world aggregate data suggests that TCI such as tacrolimus and pimecrolimus are not associated with any significant lymphoma safety signals following tabulation of the EBGM. This study highlights the importance of robust, although more complex, Bayesian analysis in determining the safety signals of rare ADRs.^3^ Recent literature has also supported a lack of lymphomagenic potential associated with TCIs.^4^^,^^5^ While gold-standard experimentation methods such as randomized controlled trials are required to confidently disbar the TCI-lymphoma association, this study may help provide reassurance to concerned clinicians in light of related-mounting evidence supporting TCI safety.

## Conflicts of interest

None disclosed.
